# Racial Disparities in Candidates for Hepatocellular Carcinoma Liver Transplant After 6-Month Wait Policy Change

**DOI:** 10.1001/jamanetworkopen.2023.41096

**Published:** 2023-11-02

**Authors:** Behnam Saberi, Ahmet Gurakar, Hani Tamim, Carolin V. Schneider, Omar T. Sims, Alan Bonder, Zachary Fricker, Saleh A. Alqahtani

**Affiliations:** 1 Beth Israel Deaconess Medical Center, Harvard Medical School, Boston, Massachusetts; 2 Johns Hopkins University, Division of Gastroenterology and Hepatology, Baltimore, Maryland; 3 College of Medicine, Alfaisal University, Riyadh, Saudi Arabia; 4 Department of Internal Medicine III, Rheinisch Westfälisch Technische Hochschule Aachen University, Aachen, Germany; 5 Department of Gastroenterology, Hepatology and Nutrition, Cleveland Clinic, Cleveland, Ohio; 6 Department of Quantitative Health Sciences, Cleveland Clinic, Cleveland, Ohio; 7 Liver Transplant Center, King Faisal Specialist Hospital, Riyadh, Saudi Arabia

## Abstract

**Importance:**

Racial disparities in liver transplant (LT) for hepatocellular carcinoma (HCC) may be associated with unequal access to life-saving treatment.

**Objective:**

To quantify racial disparities in LT for HCC and mortality after LT, adjusting for demographic, clinical, and socioeconomic factors.

**Design, Setting, and Participants:**

This cohort study was a retrospective analysis of United Network Organ Sharing/Organ Procurement Transplant Network (OPTN) data from 2003 to 2021. Participants were adult patients with HCC on the LT waiting list and those who received LT. Data were analyzed from March 2022 to September 2023.

**Exposures:**

Race and time before and after the 2015 OPTN policy change.

**Main Outcomes and Measures:**

Proportion of LT from wait-listed candidates, the proportion of waiting list removals, and mortality after LT.

**Results:**

Among 12 031 patients wait-listed for LT with HCC (mean [SD] age, 60.8 [7.4] years; 9054 [75.3%] male; 7234 [60.1%] White, 2590 [21.5%] Latinx/o/a, and 1172 [9.7%] Black or African American), this study found that after the 2015 model of end-stage liver disease (MELD) exception policy changes for HCC (era 2), the overall proportion of LT for HCC across all races decreased while the proportion of dropouts on the LT waiting list remained steady compared with patients who did not have HCC. In Kaplan-Meier analysis, Asian patients demonstrated the lowest dropout rates in both era 1 and era 2 (1-year dropout, 16% and 17%, respectively; *P* < .001). In contrast, Black or African American patients had the highest dropout rates in era 1 (1-year dropout, 24%), but comparable dropout rates (23%) with White patients (23%) and Latinx/o/a patients in era 2 (23%). In both eras, Asian patients had the highest survival after LT (5-year survival, 82% for era 1 and 86% for era 2), while Black or African American patients had the worst survival after LT (5-year survival, 71% for era 1 and 79% for era 2). In the multivariable analysis for HCC LT recipients, Black or African American race was associated with increased risk of mortality in both eras, compared with White race (HR for era 1, 1.17; 95% CI, 1.05-1.35; and HR for era 2, 1.31; 95% CI, 1.10-1.56).

**Conclusions and Relevance:**

This cohort study of LT candidates in the US found that after the 2015 MELD exception policy change for HCC, the proportion of LT for HCC had decreased for all races. Black or African American patients had worse outcomes after LT than other races. Further research is needed to identify the underlying causes of this disparity and develop strategies to improve outcomes for HCC LT candidates.

## Key Points

**Question:**

Are there racial disparities in liver transplant (LT) for hepatocellular carcinoma (HCC) and mortality after LT after the 6-month wait policy change in 2015?

**Findings:**

In this cohort study, after the 2015 model of end-stage liver disease exception policy change, liver transplant rates for HCC decreased across all races, with Asian patients showing the best outcomes and Black or African American patients the worst outcomes after LT.

**Meaning:**

These findings suggest the reasons behind continued racial disparities in LT for HCC are still unknown and warrant further investigation to develop strategies to improve outcomes for all HCC LT candidates.

## Teaser

This cohort study evaluates racial disparities among candidates for hepatocellular carcinoma liver transplant following a 6-month wait policy change.

## Introduction

Liver transplant (LT) is a life-saving treatment for end-stage liver disease, but access to this limited resource is highly competitive.^1^ After the implementation of the Milan criteria in 1996, LT was used as the best treatment option in selected patients with hepatocellular carcinoma (HCC) and cirrhosis.^2^ For allocation of LT, the model of end-stage liver disease (MELD) score is used, a numerical value based on creatinine, international normalization ratio (INR), and bilirubin, ranging from 6 to 40.^3^ MELD was designed to prioritize LT according to the urgency of the patient’s condition, as opposed to the length of time on the waiting list. However, certain liver diseases, such as HCC, may not always be associated with a high MELD score despite the severity of the disease.^4^ This discrepancy can disadvantage these patients in the organ allocation process because their MELD scores may not reflect the true urgency of their need for LT.

To achieve equity among LT candidates, in 2002, exception points were applied for patients with HCC, which led to an increase in LT for HCC.^5^ Some studies show that patients with HCC might have been overprioritized during that period.^6^ Therefore, in October 2015, the revised Organ Procurement and Transplantation Network (OPTN) policy introduced a 6-month delay before a MELD score of 28 could be assigned and restricted the maximum HCC exception score to 34 points.^7^ Although some studies have shown that prolonging HCC waiting list times by up to 6 months improved equity among LT candidates according to their HCC status, its association with survival after LT and race, in particular, is not well characterized.^8,9^

Despite efforts to reduce disparities in LT, such as the Share 35 policy,^10^ split liver transplant, or the increasing utilization of living donor LTs, there is still a significant gap in access and outcomes among different racial groups.^4,11,12,13^ It is well known that Latinx/o/a and Black or African American patients are less frequently referred to LT centers and have a lower chance of getting on the waiting list for LT.^14,15,16^ Moreover, especially in the case of HCC, it is known that Black or African American and Latinx/o/a patients are often diagnosed at more advanced disease stages and have lower rates of living donor LT and HCC resection.^2,17^

Still, there are limited recent data on characteristics, outcomes, and predictors of survival in patients with HCC undergoing LT according to race, particularly following the 2015 MELD exception policy change. This study aimed to investigate the association between race and dropouts on the LT waiting list and mortality after LT in the US, using a large national database of adult LT candidates.

## Methods

### Data Source

The data reported were supplied by the United Network for Organ Sharing (UNOS), as the contractor for the OPTN. The interpretation and reporting of these data are the responsibility of the author(s) and should not be seen as an official policy or interpretation by the OPTN or the US government. Because UNOS is a publicly available, deidentified, patient-level database, informed consent and ethical approval were not required according to the policies of UNOS and the institutional review board at Beth Israel Deaconess Medical Center. This study followed the Strengthening the Reporting of Observational Studies in Epidemiology (STROBE) reporting guideline.

### Study Design and Patient Population

This was a retrospective cohort study using the UNOS database. Analyses of LT waiting list dropout included all adult (aged >18 years) LT candidates wait-listed between January 2010 and December 2021. Dropout was defined as removal from the transplant waiting list without receiving a transplant. These reasons include deterioration of health as too ill to transplant or death while waiting. Analyses of survival after LT included all adult (aged >18 years) recipients who underwent deceased donor liver transplant (DDLT) from January 2010 through December 2021. For survival analysis, we categorized this period into 2 eras (era 1, 2010 to 2015, and era 2, 2016 to 2021).

### Recipient and Waiting List Candidate Characteristics

Recipient and waiting list candidate demographic data included were age, sex (male or female), and race (Asian, Black or African American, Latinx/o/a, and White). The race information was self-reported and provided through the UNOS database.^18^ Patients whose race was reported as other or unclear were excluded from our study. Clinical characteristics included the cause of liver disease (hepatitis C virus [HCV], hepatitis B virus [HBV], alcoholic liver disease [ALD] and non-alcoholic fatty liver disease [NAFLD], blood type [A, B, AB, or O]), and various clinical characteristics (HCC, diabetes, ascites, hepatic encephalopathy, or dialysis). Laboratory data included were albumin, creatinine, total bilirubin, INR, calculated MELD score, and MELD exception.

### Outcome

The primary outcome was survival after LT in eras 1 and 2. The secondary outcome was liver transplant waiting list dropout (too ill to transplant or died). In addition, we calculated the proportion of LT and dropouts among all candidates on the LT waiting list, among all removal reasons such as transplanted, condition improved, unable to contact the patient, medically unsuitable, too ill to transplant, or deceased. It is important to note that the proportions were calculated according to all removal reasons reported in the UNOS/OPTN site. These proportions were not calculated according to waiting list additions for each year or the total number of waiting list candidates at the beginning or end of the year.

### Statistical Analysis

Data were extracted from the database and transferred into SAS, version 9.4 (SAS Institute) and STATA, version 16.1 (StataCorp) for data cleaning, management, and analyses by 2 independent statisticians (H.T. and B.S.). This was done to confirm the accuracy of the results. Demographic and clinical characteristics were summarized. Categorical variables are presented as numbers and percentages; continuous variables are reported as mean and standard deviation. Characteristics were reported for the total study population and compared across the category of racial background, using the *t* test for continuous variables and the χ^2^ test for categorical variables. Associations of characteristics with race were evaluated separately in wait-listed candidates and DDLT recipients and were further stratified by HCC status.

Kaplan-Meier analysis was used to evaluate time to event. The time to death or dropout was used as our time variable. The log-rank test was used to evaluate the statistical significance of potential differences in survival after LT distributions across races. Those who were lost to follow-up were censored in the survival analyses. As a sensitivity analysis, we carried out an additional Kaplan-Meier curve analysis to evaluate dropouts on the LT waiting list that included all wait-listed patients who received or did not receive LT and LT acting as a competing risk. For the dropout analysis, patients who were lost to follow-up or received an LT were censored.

To evaluate the association between race and mortality in LT recipients while accounting for important potential confounders, we used a stepwise multivariable Cox proportional hazards model. Variables included in the model were donor and recipient variables that showed statistical significance at the bivariable level. For the recipient, age was calculated in 10-year intervals. These intervals were selected to yield clinically meaningful hazard ratios (HRs). Additionally, a competing risk analysis was performed. We assessed the interaction between race and era in the multivariable analysis, and we reported the *P* value for this interaction. A cutoff *P*-value of .05 was used to include and exclude the variables from the final model. Results are presented as HR and 95% CI. Throughout the study, *P* ≤ .05 was considered statistically significant. Data were analyzed from March 2022 to September 2023.

## Results

### Characteristics of Wait-Listed Candidates by Race

We analyzed 12 031 wait-listed patients. Their mean (SD) age was 60.8 (7.4) years, 9054 [75.3%] were male; 7234 [60.1%] were White, 2590 [21.5%] Latinx/o/a, and 1172 [9.7%] Black or African American. Demographic and clinical characteristics of waiting list candidates with HCC who did not receive LT, stratified by race, are summarized in Table 1, and patients without HCC are shown in eTable 1 in Supplement 1 for comparison. Asian candidates had a lower body mass index than other races. HCV was more common in Black or African American patients, HBV was more common in Asian patients, and ALD and NAFLD were more common in White and Latinx/o/a patients. Diabetes was more common among Latinx/o/a candidates compared with other races. Hepatic encephalopathy and ascites were more common in White and Latinx/o/a patients.

**Table 1.** Characteristics of Liver Transplant Waiting List Candidates With Hepatocellular Carcinoma According to Race^a^

Characteristic	Participants, No. (%)				
	Total (N = 12 031)	Asian (n = 1035)	Black or African American (n = 1172)	Latinx/o/a (n = 2590)	White (n = 7234)
Age, mean (SD), y	60.8 (7.4)	60.5 (8.3)	60.3 (7.4)	60.3 (7.6)	61.2 (7.1)
Sex					
Female	2977 (24.7)	259 (25.0)	333 (28.4)	754 (29.1)	1631 (22.6)
Male	9054 (75.3)	776 (75.0)	839 (71.6)	1836 (70.9)	5603 (77.4)
Blood type					
A	4470 (37.2)	271 (26.2)	322 (27.5)	800 (30.9)	3077 (42.5)
B	1352 (11.2)	269 (26.0)	206 (17.6)	219 (8.5)	658 (9.1)
AB	308 (2.6)	56 (5.4)	41 (3.5)	32 (1.2)	179 (2.5)
O	5901 (49.1)	439 (42.4)	603 (51.5)	1539 (59.4)	3320 (45.9)
Body mass index, mean (SD)^b^	28.9 (5.6)	25.2 (4.1)	28.5 (5.5)	29.9 (5.6)	29.2 (5.6)
Cause					
HCV	5733 (47.7)	298 (28.8)	803 (68.5)	1090 (42.1)	3542 (49.0)
ALD	1591 (13.2)	39 (3.8)	40 (3.4)	483 (18.7)	1029 (14.2)
NAFLD	1573 (13.1)	48 (4.6)	12 (1.0)	462 (17.8)	1051 (14.5)
HBV	544 (4.5)	366 (35.4)	63 (5.4)	23 (0.9)	92 (1.3)
Other	2590 (21.5)	284 (27.4)	254 (21.7)	532 (20.5)	1520 (21.0)
Diabetes	4131 (34.3)	351 (33.9)	392 (33.5)	1099 (42.4)	2289 (31.6)
Hepatic encephalopathy	4222 (35.1)	163 (15.8)	316 (27.0)	970 (37.5)	2773 (38.3)
Ascites	5055 (42.0)	230 (22.2)	419 (35.8)	1132 (43.7)	3274 (45.3)
MELD Score, mean (SD)	15.4 (9.5)	12.5 (8.4)	14.7 (9.3)	16.5 (9.7)	15.6 (9.5)
MELD exception, mean (SD)	17.1 (10.1)	15.4 (10.0)	15.9 (9.9)	17.8 (10.4)	17.3 (10.0)
Creatinine, mean (SD), mg/dl	1.3 (1.2)	1.2 (1.0)	1.5 (1.7)	1.2 (1.1)	1.2 (1.1)
Bilirubin, mean (SD), mg/dL	3.9 (7.2)	3.0 (6.5)	3.4 (6.9)	4.4 (7.7)	4.0 (7.1)
INR, mean (SD)	1.6 (1.1)	1.4 (0.9)	1.5 (1.0)	1.6 (1.2)	1.6 (1.1)
Albumin, mean (SD), g/dL	3.3 (0.8)	3.6 (0.8)	3.3 (0.8)	3.2 (0.7)	3.3 (0.7)
Wait time, mean (SD), d	342.1 (513.7)	401.5 (520.6)	295.7 (429.9)	402.0 (578.7)	327.2 (504.4)

Abbreviations: ALD, alcoholic liver disease; HBV, hepatitis B virus; HCV, hepatitis C virus; INR, international normalization ratio; MELD, model for end-stage liver disease; NAFLD, non-alcoholic fatty liver disease.

SI Conversion factors: To convert albumin to grams per liter, multiply by 10; bilirubin to micromoles per liter, multiply by 17.104; creatinine to micromoles per liter, multiply by 88.4.

^a^ Patients who identified as other race are not included in this table.

^b^ Body mass index is calculated as weight in kilograms divided by height in meters squared.

### Trends in DDLT and Dropout for HCC Candidates

The eFigure in Supplement 1 shows the overall trend in the proportion of LT and dropout among HCC waiting list candidates by race between 2003 and 2021. The proportion of LT and dropouts (too ill to transplant or deceased) was calculated among all candidates removed from the LT waiting list for each year. For HCC, there was a decrease in the proportion of LT recipients after 2015 for all races (eFigure in Supplement 1). In comparison, for patients without HCC, there was an increase in the proportion of LT recipients after 2015 for all races and a decrease in dropouts after 2015 for all races (eFigure in Supplement 1).

**Figure 1.**
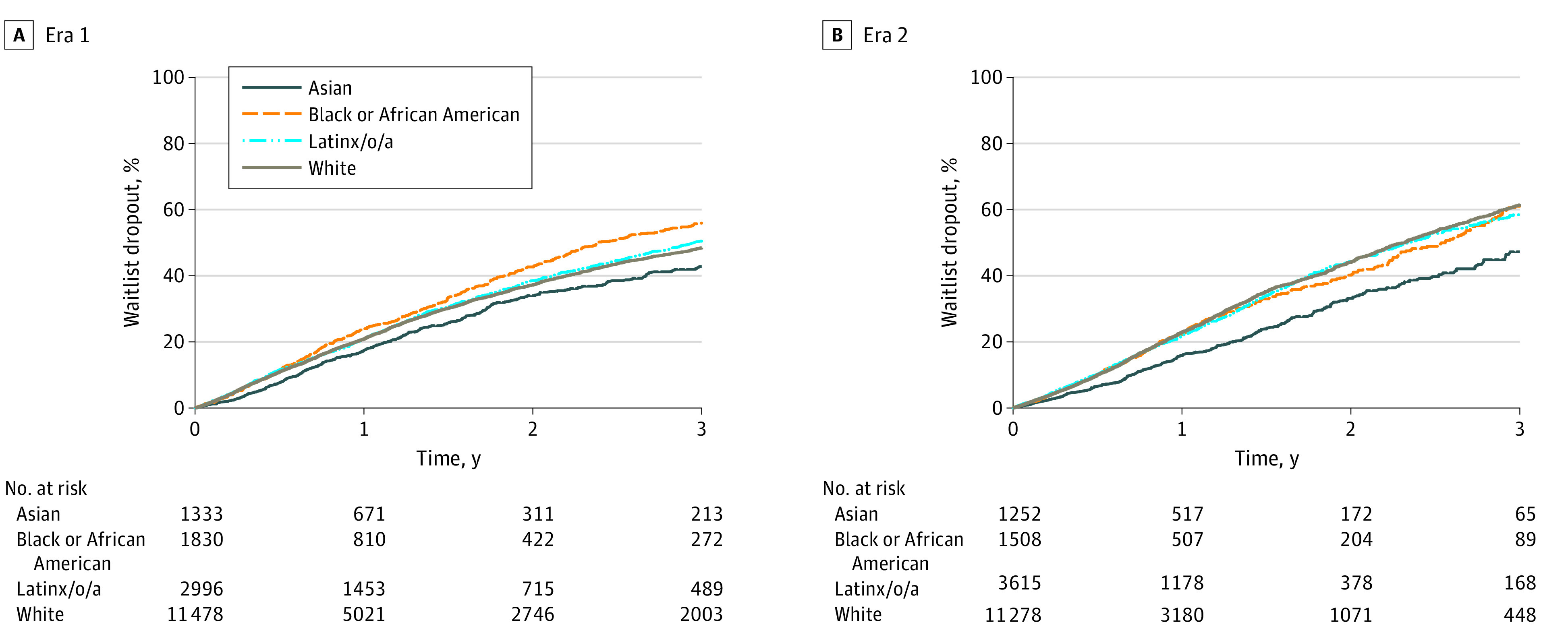
Kaplan-Meier Curve Analysis for Time to Dropout for Hepatocellular Carcinoma Patients on the Waiting List *P* value for both eras <.001.

We performed a Kaplan-Meier curve in all wait-listed patients who received or did not receive a transplant, where time to dropout was analyzed in era 1 and era 2. Asian patients showed the lowest dropout rates in era 1 and era 2 (1-year dropout, 16% and 17%, respectively; *P* < .001) (Figure 1A, Figure 1B; eTable 2 and eTable 3 in Supplement 1). Black or African American patients had the highest dropout rate in era 1 compared with other races (1-year dropout, 24%) (Figure 1A**;** eTable 2 in Supplement 1). In era 2, Black or African American patients had similar dropout rates compared with White and Latinx/o/a patients (all 23%) (Figure 1B**;** eTable 3 in Supplement 1).

### Characteristics of DDLT Recipients According to Race

Demographic and clinical characteristics of LT recipients with HCC, stratified by race, are summarized in Table 2. Regardless of HCC status, Black or African American patients were more likely to be female (333 patients [28.4%]) and have a B blood type (206 patients [17.6%]). HCV was more common in Black or African American patients (803 patients [68.5%]) and HBV was more common in Asian patients (366 patients [35.4%]), while ALD and NAFLD were more common in White and Latinx/o/a patients. Diabetes was more prevalent in the Latinx/o/a LT recipients (1099 patients [42.4%]) compared with the other races. The wait time was longest for Asian and Latinx/o/a patients (mean [SD] 402 [520] days, compared with 327 [504] days for White patients and 296 [430] days for Black or African American patients; *P* < .001). For comparison, the demographics and clinical characteristics of LT recipients without HCC are summarized in eTable 4 in Supplement 1.

**Table 2.** Characteristics of Hepatocellular Carcinoma Deceased Donor Liver Transplant Recipients According to Race

Characteristic	Participants, No. (%)				
	Total (N = 23 080)	Asian (n = 1565)	Black or African American (n = 2179)	Latinx/o/a (n = 3949)	White (n = 15 387)
Recipient characteristics					
Age, mean (SD), y	60.2 (7.3)	59.7 (8.2)	59.7 (7.9)	59.6 (7.4)	60.5 (7.1)
Sex					
Female	5347 (23.2)	391 (25.0)	623 (28.6)	1088 (27.6)	3245 (21.1)
Male	17 733 (76.8)	1174 (75.0)	1556 (71.4)	2861 (72.4)	12 142 (78.9)
Blood type					
A	8495 (36.8)	433 (27.7)	550 (25.2)	1240 (31.4)	6272 (40.8)
B	3151 (13.7)	480 (30.7)	484 (22.2)	431 (10.9)	1756 (11.4)
AB	1069 (4.6)	139 (8.9)	102 (4.7)	98 (2.5)	730 (4.7)
O	10 365 (44.9)	513 (32.8)	1043 (47.9)	2180 (55.2)	6629 (43.1)
Body mass, mean (SD)^a^	29.2 (5.3)	25.5 (3.8)	28.9 (5.3)	29.6 (5.2)	29.5 (5.3)
Cause					
HCV	13 735 (59.5)	540 (34.5)	1761 (80.8)	2187 (55.4)	9247 (60.1)
ALD	2678 (11.6)	54 (3.5)	71 (3.3)	698 (17.7)	1855 (12.1)
NAFLD	2871 (12.4)	84 (5.4)	25 (1.2)	548 (13.9)	2214 (14.4)
HBV	1247 (5.4)	771 (49.3)	141 (6.5)	47 (1.2)	288 (1.9)
Other	2549 (11.0)	116 (7.4)	181 (8.3)	469 (11.9)	1783 (11.6)
Diabetes	7831 (33.9)	522 (33.4)	695 (31.9)	1630 (41.3)	4984 (32.4)
Hepatic encephalopathy	10 317 (44.7)	370 (23.6)	771 (35.4)	1941 (49.2)	7235 (47.0)
Ascites	13 162 (57.0)	561 (35.9)	1081 (49.6)	2412 (61.1)	9108 (59.2)
MELD Score, mean (SD)	15.6 (8.8)	12.7 (8.1)	15.3 (8.9)	17.3 (9.7)	15.6 (8.5)
MELD exception, mean (SD)	27.2 (5.7)	28.4 (5.3)	27.0 (5.9)	28.6 (6.1)	26.8 (5.6)
Creatinine, mean (SD), mg/dl	1.2 (1.0)	1.1 (0.9)	1.4 (1.5)	1.2 (1.1)	1.1 (0.8)
Bilirubin, mean (SD), mg/dL	3.8 (6.8)	3.0 (6.7)	3.4 (6.4)	4.9 (8.0)	3.7 (6.5)
INR, mean (SD)	1.5 (0.8)	1.4 (0.7)	1.5 (0.9)	1.6 (0.8)	1.5 (0.8)
Albumin, mean (SD), g/dL	3.3 (0.7)	3.6 (0.7)	3.2 (0.7)	3.2 (0.7)	3.3 (0.7)
Wait time, mean (SD), d	342.1 (513.7)	401.5 (520.6)	295.7 (429.9)	402.0 (578.7)	327.2 (504.4)
Donor characteristics					
Age, mean (SD), y	42.8 (16.3)	43.0 (17.8)	42.2 (15.8)	42.7 (16.6)	42.9 (16.2)
Sex					
Male	13 913 (60.3)	838 (53.6)	1287 (59.1)	2313 (58.6)	9475 (61.6)
Female	9167 (39.7)	727 (46.5)	892 (40.9)	1636 (41.4)	5912 (38.4)
Body mass index, mean (SD)^a^	28.2 (6.6)	26.7 (5.9)	28.2 (6.7)	27.8 (6.3)	28.5 (6.7)
Race					
Asian	598 (2.6)	124 (7.9)	43 (2.0)	132 (3.3)	299 (1.9)
Black or African American	4069 (17.6)	278 (17.8)	517 (23.7)	598 (15.1)	2676 (17.4)
Latinx/o/a	3112 (13.5)	261 (16.7)	227 (10.4)	928 (23.5)	1696 (11.0)
White	14 901 (64.6)	863 (55.1)	1372 (63.0)	2188 (55.4)	10 478 (68.1)
Total cold ischemic time, mean (SD), h	6.2 (2.4)	6.5 (2.5)	6.0 (2.3)	6.4 (2.5)	6.2 (2.4)

Abbreviations: ALD, alcoholic liver disease; HBV, hepatitis B virus; HCV, hepatitis C virus; INR, international normalization ratio; MELD, model for end-stage liver disease; NAFLD, non-alcoholic fatty liver disease.

SI Conversion factors: To convert albumin to grams per liter, multiply by 10; bilirubin to micromoles per liter, multiply by 17.104; creatinine to micromoles per liter, multiply by 88.4.

^a^ Body mass index is calculated as weight in kilograms divided by height in meters squared.

### Post-DDLT Survival According to Race

**Figure 2.**
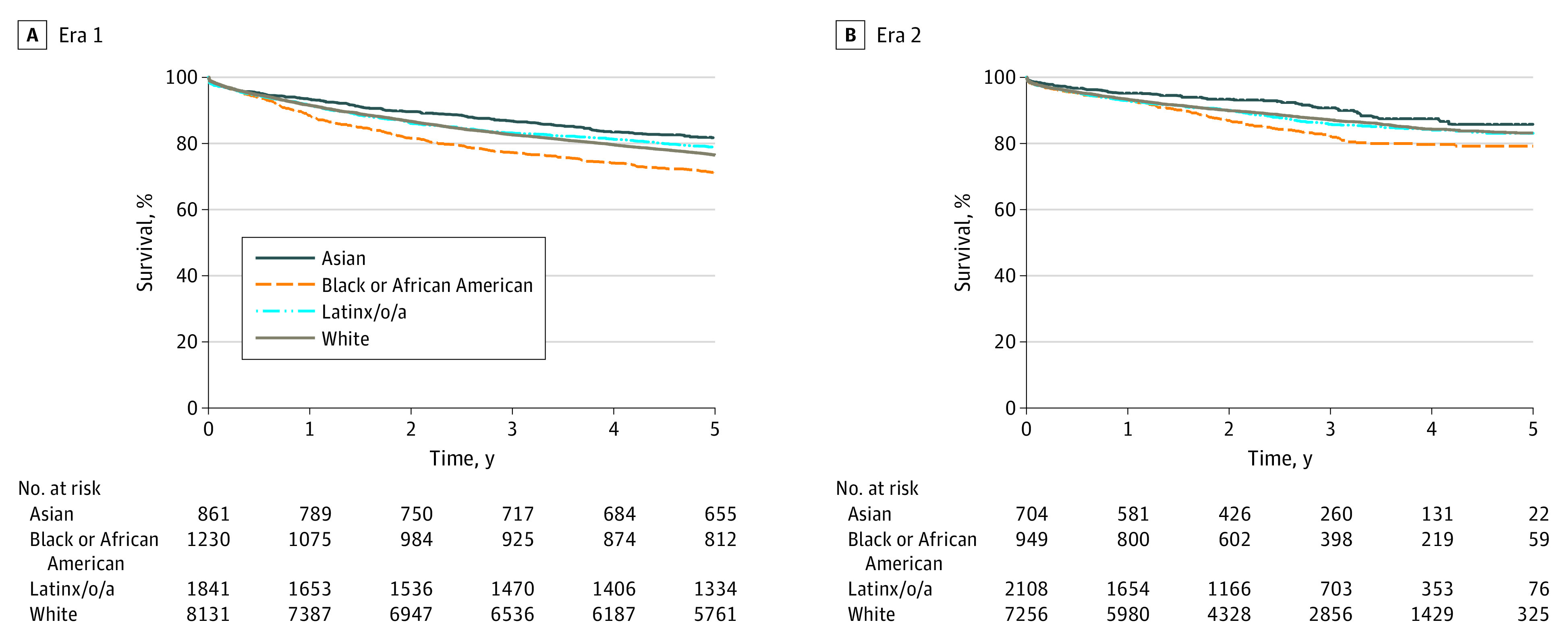
Survival Among Hepatocellular Carcinoma Liver Transplant Recipients According to Race *P* value for both eras <.001.

Figure 2 shows Kaplan-Meier survival after LT in HCC stratified by race. Analyses were conducted separately according to the period. In LT recipients with HCC, Asian patients had the longest survival after LT, regardless of the period (5-year survival, 82% in era 1 and 86% in era 2). Black or African American patients had the worst survival after LT in both eras 1 and 2 (5-year survival, 71% and 79%, respectively; *P* < .001) (Figure 2; eTable 5 and eTable 6 in Supplement 1). In era 1, Latinx/o/a patients had superior survival after LT compared with Black or African American and White patients (Figure 2A; eTable 5 in Supplement 1). In era 2, White and Latinx/o/a patients had comparable survival (Figure 2B**;** eTable 6 in Supplement 1).

### Multivariable Analysis of LT Recipients With HCC

Table 3 shows factors associated with mortality in LT recipients in the HCC group in different eras. Variables that were associated with mortality in both eras included older recipient and donor age, male sex, encephalopathy, diabetes, higher MELD, higher creatinine, and lower albumin. Overall, era 2 was associated with improved survival (HR, 0.72; 95% CI, 0.67-0.77) compared with era 1. In era 1, Black or African American race was associated with increased risk of mortality compared with White race (HR, 1.17; 95% CI, 1.05-1.35), while Latinx/o/a and Asian race were associated with lower mortality compared with White race (HR, 0.58; 95% CI, 0.77-0.93 and HR, 0.87; 95% CI, 0.74-1.02, respectively). In era 2, Black or African American race was associated with higher mortality compared with White race (HR, 1.31; 95% CI, 1.10-1.56). We additionally included blood type in a repeated multivariable analysis, but the results did not differ. When looking at the interaction in terms of race and era, in era 2, there was no improvement for Black or African American patients’ survival after LT as compared with White patients. In contrast, there was a significant decrease for survival after LT for the Latinx/o/a group between era 1 and era 2 as compared with White patients (*P* for interaction = .03) (Table 3).

**Table 3.** Multivariable Cox Proportional Hazard Analysis for the Association Between Race and Mortality Among Hepatocellular Carcinoma (HCC) LT Recipients by Era^a^

All patients			Era 1		Era 2	*P* value	
Characteristic	HR (95% CI)	*P* value	HR (95% CI)	*P* value	HR (95% CI)	Era 2	Interaction of race and era
Race (White as reference)							
Asian	0.89 (0.77-1.02)	.09	0.87 (0.74-1.02)	.09	0.84 (0.65-1.08)	.18	.93
Black or African American	1.22 (1.11-1.33)	<.001	1.17 (1.05-1.30)	.003	1.31 (1.10-1.56)	.003	.37
Latinx/o/a	0.90 (0.83-0.98	.01	0.85 (0.77-0.93)	<.001	1.04 (0.90-1.19)	.61	.03
Era	0.72 (0.67-0.77)	<.001	NA	NA	NA	NA	NA
Recipient characteristics							
Age, y	1.25 (1.20-1.31)	<.001	1.27 (1.21-1.34)	<.001	1.24 (1.14-1.34)	<.001	NA
Sex	0.91 (0.84-0.97)	.005	NA	NA	NA	NA	NA
Diabetes	1.19 (1.12-1.27)	<.001	1.23 (1.14-1.32)	<.001	NA	NA	NA
Hepatic encephalopathy	1.19 (1.12-1.26)	<.001	1.19 (1.11-1.27)	<.001	1.18 (1.06-1.32)	.003	NA
Creatinine, mg/dl	1.08 (1.05-1.10)	<.001	1.08 (1.05-1.11)	<.001	1.09 (1.04-1.14)	<.001	NA
Albumin, g/dL	0.89 (0.85-0.93)	<.001	0.92 (0.87-0.96)	<.001	0.84 (0.78-0.91)	<.001	NA
MELD	1.01 (1.00-1.01)	.03	NA	NA	NA	NA	NA
Cause							
ALD	0.84 (0.76-0.93)	<.001	0.84 (0.74-0.95)	.005	NA	NA	NA
NAFLD	0.90 (0.82-0.99)	.03	0.80 (0.71-0.91)	<.001	NA	NA	NA
HBV	0.77 (0.65-0.90)	.001	0.75 (0.62-0.90)	.002	NA	NA	NA
Donor characteristics							
Age, median (IQR), y	1.05 (1.04-1.07)	<.001	1.06 (1.04-1.0)8	<.001	NA	NA	NA

Abbreviations: ALD, alcoholic liver disease; HBV, hepatitis B virus; HR, hazard ratio; MELD, model for end-stage liver disease; NAFLD, non-alcoholic fatty liver disease.

^a^ Variables included in the model, recipient characteristics: age, sex (reference: male), race (reference: White), era (reference: era 1), diabetes, HE, ascites, transjugular intrahepatic shunt, MELD, sodium, creatinine, albumin, wait time, length of hospital stay after transplant; donor characteristics: age.

## Discussion

The present study aimed to assess racial disparities in LT waiting list dropout rates and mortality after LT following the 2015 MELD exception policy change. Our findings reveal persistent disparities in survival after LT and waiting list outcomes among racial groups in the US. Several notable findings emerged from our study. First, after the 2015 MELD exception policy change (era 2), the rate of LT was decreasing, and the rate of dropout was steady for HCC in all races relative to patient who did not have HCC. In regards to dropout, the Kaplan Meier analysis showed that Asian patients had the lowest dropout in era 1 and 2 compared with other races. In era 1, Black or African American patients had the highest dropout. However, after the 2015 MELD exception policy, this changed, and they showed similar dropout compared with White and Latinx/o/a patients. Regarding survival after LT, Black or African American patients showed the lowest survival, and Asian patients showed the highest survival in both eras.

Our multivariable models reveal that Black or African American patients had the lowest survival after LT, regardless of HCC status and across both eras. These findings align with a former UNOS study that analyzed data until 2017,^19^ but that study additionally reported a decreasing difference over time, which is not seen in our study after 2015. It is unclear why Black or African American patients have the highest mortality after transplant, irrespective of the era. Survival after liver transplant is complex as it relates to all-cause mortality. Some of the most common causes of death following liver transplant in HCC include heart disease, cancers, and graft failure.^20^ Patients are also at risk of diabetes, hypertension, dyslipidemia, and malignant neoplasm after LT.^21^ Therefore, monitoring and care following transplant is critical to screen and treat patients with these conditions. It is also important to note that HCV is the most common cause of liver disease in Black or African American patients with HCC LT. Studying the causes of death after LT according to race, access to care, screening, and HCV treatment are all important areas of investigation for future research. Interestingly, dropout is improved in era 2 for all races compared with era 1. This should be an area of future research. Still, all patients with HCC appear to face disadvantages following the 2015 OPTN policy. Attributing the decline in LT rates and higher dropout rates for patients with HCC solely to the 2015 MELD exception policy change is challenging. Our results suggest that specific and increasing barriers faced by minoritized populations are not effectively addressed by current policies and should be the focus of future studies.^19,22^ We hypothesize that Asian patients show the lowest dropout and higher survival rate because they have more HBV, the lowest MELD scores, and were not as often decompensated. But underlying causes of marked differences in survival after LT for all racial groups are likely due to social determinants of health.^23,24^ Both patient access and patient survival are largely influenced by social determinants of health. Addressing health disparities requires considering both upstream and downstream factors.^25^ Upstream efforts involve systemic changes and social interventions to promote equitable access to care. Downstream interventions focus on individual behaviors and delivery improvements.^26^ According to prior studies, social deprivation (ie, area-level^27^ and neighborhood-level determinants^28^), economic stability,^29^ education attainment,^14^ income,^30^ sex,^31,32^ insurance status (public vs private), and cost^33^ are a few plausible factors that disadvantage patients on LT waiting lists and after LT.^34^ Accordingly, there is a strong need for future research to develop strategies to counter health inequities that contribute to limited access^35^ to care and address cultural and linguistic barriers that may prevent certain racial groups from accessing LT.^36^

In recent years, there have been significant efforts to address and reduce disparities in LT.^37^ In June 2013, the UNOS implemented the Regional Share 35 policy to expand regional liver donor sharing according to acuity of illness rather than distance in an effort to reduce geographic disparities.^10^ Several studies have since explored strategies aimed at mitigating disparities.^38^ Cholankeril et al^39^ studied how multiple listings in UNOS regions contribute to inequities. Ye et al^40^ hypothesized that raising the minimum MELD threshold for local allocation decreases MELD variance without significant effects on waiting list deaths. Additionally, initiatives such as the Minority Organ and Tissue Transplant Education Program focus on increasing donation and transplantation among minority populations through community education and outreach.^41^ These previous efforts serve as a foundation for ongoing research.

As a result of our findings, we see the urgent need to address racial disparities in liver transplant access and outcomes through a multifaceted approach.^42^ This involves investigating the primary roots of these findings. For example, one such root is understanding the causes of death and dropout in various races according to geographic locations throughout the US, in patients before or after LT. We may be able to address the challenges and suggest policy changes based on this information.

### Limitations and Strengths

The study presented here, while providing valuable insights, is not without limitations. One important limitation is that the racial classification used in the analysis was based on the codes available in the database. Although efforts were made to accurately classify patients according to these codes, there may be inherent limitations and potential misclassification. Additionally, given the observational study design, it is crucial to acknowledge that causal relationships cannot be assumed due to the inherent restraints of this design.

Furthermore, the analysis was constrained by the variables available in the OPTN/UNOS database. Although the study focused on important factors related to LT, there may be other variables of interest that were not available for inclusion in the analysis. These unmeasured variables could potentially have an impact on survival after liver transplant and introduce additional confounding factors that were not accounted for in this study. For instance, factors such as tumor number and size, α-fetoprotein levels, and other clinical variables related to HCC were not included in this analysis, which may limit a comprehensive understanding of the association between these factors and survival outcomes.

Despite these limitations, the study exhibits several notable strengths. First, it used very few patient exclusion criteria, allowing for a broad representation of the population. Coupled with the extended study period and a large and diverse sample size, this study contributes to the generalizability of the findings to the broader US population of LT waiting list candidates and recipients. Second, the study incorporated a wide range of patient demographic and clinical characteristics in statistical modeling and diligently controlled for important confounding factors, including age, sex, and MELD score. This comprehensive approach enhances the robustness and validity of the findings.

## Conclusions

Our study provides important insights into the association between the 2015 MELD exception policy change for HCC, waiting list dynamics, and survival after transplant across different racial groups. Our findings reveal a notable decrease in the proportion of LT on the waiting list for HCC among all races following the policy change. These findings contribute to the existing knowledge and understanding of factors influencing liver transplant outcomes, albeit within the constraints of the available data and study design. Furthermore, transplant survival after transplant for HCC was found to be lowest among Black or African American patients, with Asian patients demonstrating the highest survival rates. Ultimately, achieving health equity in the field of LT necessitates a comprehensive approach that considers the intricate interplay of social, cultural, and systemic factors.

## Notes

Supplement 1. **eTable 1. **Characteristics of Wait-Listed Candidates Who Did Not Have HCC According to Race **eTable 2. **Comparison of Dropout in Patients With HCC in Era 1 According to Race **eTable 3. **Comparison of Dropout in Patients With HCC in Era 2 According to Race **eTable 4. **Characteristics of LT Recipients Who Did Not Have HCC According to Race **eTable 5. **Comparison of Post-LT Survival in Patients With HCC in Era 1 According to Race **eTable 6. **Comparison of Post-LT Survival in Patients With HCC in Era 2 According to Race **eFigure. **Trend of Proportion of Liver Transplants

Supplement 2.
Data Sharing Statement
