# Supplementary material for: Racial Disparities in Candidates for Hepatocellular Carcinoma Liver Transplant After 6-Month Wait Policy Change
Source: JAMA Netw Open. 2023 Nov 2;6(11):e2341096. doi: 10.1001/jamanetworkopen.2023.41096 (PMC10623194; doi:10.1001/jamanetworkopen.2023.41096)
Supplement: Supplement 1. — eTable 1. Characteristics of Wait-Listed Candidates Who Did Not Have HCC According to Race eTable 2. Comparison of Dropout in Patients With HCC in Era 1 According to Race eTable 3. Comparison of Dropout in Patients With HCC in Era 2 According to Race eTable 4. Characteristics of LT Recipients Who Did Not Have HCC According to Race eTable 5. Comparison of Post-LT Survival in Patients With HCC in Era 1 According to Race eTable 6. Comparison of Post-LT Survival in Patients With HCC in Era 2 According to Race eFigure. Trend of Proportion of Liver Transplants [file jamanetwopen-e2341096-s001.pdf]

Supplemental Online Content

Saberi B, Gurakur A, Tamim H, et al. Racial disparities among HCC liver transplant candidates following 6-month wait policy change. *JAMA Netw Open*. 2023;6(11):e2341096. doi:10.1001/jamanetworkopen.2023.41096

eTable 1. Characteristics of Waitlisted Candidates Who Did Not Have HCC According to Race
eTable 2. Comparison of Dropout in Patients With HCC in Era 1 According to Race
eTable 3. Comparison of Dropout in Patients With HCC in Era 2 According to Race
eTable 4. Characteristics of LT Recipients Who Did Not Have HCC Accoridng to Race
eTable 5. Comparison of Post-LT Survival in Patients With HCC in Era 1 According to Race
eTable 6. Comparison of Post-LT Survival in Patients With HCC in Era 2 According to Race
eFigure. Trend of Proportion of Liver Transplants

This supplemental material has been provided by the authors to give readers additional information about their work.

© 2023 Saberi B et al. *JAMA Network Open.*

eTable 1. Characteristics of Waitlisted Candidates Who Did Not Have HCC According to Race

Characteristics	Total* N=36936	Asian N=1207	Black/AA N=2545	Latinx/o/a N=6387	White N=26797	p-value
**Age, Mean (SD)**	54.8 (10.7)	53.5 (11.8)	52.1 (12.2)	54.3 (10.6)	55.2 (10.5)	<.0001
**Female, n (%)**	15826 (42.9%)	495 (41.0%)	1256 (49.4%)	2841 (44.5%)	11234 (41.9%)	<.0001
**Blood type, n (%)**						<.0001
A	14560 (39.4%)	350 (29.0%)	711 (27.9%)	1946 (30.5%)	11553 (43.1%)	
B	3809 (10.3%)	347 (28.8%)	438 (17.2%)	574 (9.0%)	2450 (9.1%)	
AB	882 (2.4%)	75 (6.2%)	75 (3.0%)	92 (1.4%)	640 (2.4%)	
O	17685 (47.9%)	435 (36.0%)	1321 (51.9%)	3775 (59.1%)	12154 (45.4%)	
**BMI (kg/m^2), Mean (SD)**	28.8 (6.2)	25.7 (5.1)	28.5 (6.2)	29.2 (6.1)	28.8 (6.2)	<.0001
**Etiology, n (%)**						<.0001
1 (HCV)	7659 (20.7%)	178 (14.8%)	850 (33.4%)	1413 (22.1%)	5218 (19.5%)	
2 (ALD)	12312 (33.3%)	233 (19.3%)	437 (17.2%)	2069 (32.4%)	9573 (35.7%)	
3 (NAFLD)	6783 (18.4%)	136 (11.3%)	119 (4.7%)	1252 (19.6%)	5276 (19.7%)	
4 (HBV)	503 (1.4%)	224 (18.6%)	68 (2.7%)	30 (0.5%)	181 (0.7%)	
5 (other)	9679 (26.2%)	436 (36.1%)	1071 (42.1%)	1623 (25.4%)	6549 (24.4%)	
**Diabetes mellitus, n (%)**	10433 (28.3%)	364 (30.2%)	739 (29.0%)	2261 (35.4%)	7069 (26.4%)	<.0001
**HE, n (%)**	20770 (56.2%)	509 (42.2%)	1267 (49.8%)	3560 (55.7%)	15434 (57.6%)	<.0001
**Ascites, n (%)**	23547 (63.8%)	602 (49.9%)	1466 (57.6%)	3992 (62.5%)	17487 (65.3%)	<.0001
**MELD Score, Mean (SD)**	20.7 (10.7)	21.2 (11.6)	22.5 (11.3)	22.0 (10.9)	20.2 (10.6)	<.0001
**MELD exception, Mean (SD)**	19.9 (11.1)	20.6 (12.0)	21.7 (11.8)	20.9 (11.6)	19.5 (10.9)	<.0001
**Creatinine (mg/dl), Mean (SD)**	1.6 (1.6)	1.7 (1.9)	2.3 (2.5)	1.7 (1.8)	1.5 (1.3)	<.0001
**Bilirubin (mg/dL), Mean (SD)**	6.8 (10.1)	8.7 (12.5)	8.1 (11.1)	7.7 (11.2)	6.4 (9.6)	<.0001
**INR, Mean (SD)**	1.9 (1.4)	2.0 (1.8)	2.1 (1.7)	2.0 (1.5)	1.9 (1.4)	<.0001
**Albumin (g/dL), Mean (SD)**	3.2 (0.8)	3.3 (0.8)	3.1 (0.8)	3.1 (0.8)	3.2 (0.7)	<.0001

*Patients who identified as “Other race” are not included in this table.

Abbreviations: ALD: alcoholic liver disease; BMI: body mass index, HBV: hepatic B virus, HCC: hepatocellular carcinoma, HCV: hepatic C virus, HE: hepatic encephalopathy, INR: international normalisation ratio, MELD: model for end-stage liver disease, NAFLD: non-alcoholic fatty liver disease, PVT: portal vein thrombosis; SD: standard deviation, TIPS: transjugular intrahepatic shunt

© 2023 Saberi B et al. *JAMA Network Open.*

eTable 2. Comparison of Dropout in Patients With HCC in Era 1 According to Race

**Comparison**	**p-values**
Across all 4groups	< 0.001
White vs Black/AA	0.008
White vs Latinx/o/a	0.77
White vs Asian	<0.001
Black/AA vs Latinx/o/a	0.01
Black/AA vs Asian	<0.001
Latinx/o/a vs Asian	<0.001

© 2023 Saberi B et al. *JAMA Network Open.*

eTable 3. Comparison of Dropout in Patients With HCC in Era 2 According to Race

Comparison	p-values
Across all 4 groups	< 0.001
White vs Black/AA	0.73
White vs Latinx/o/a	0.36
White vs Asian	<0.001
Black/AA vs Latinx/o/a	0.73
Black/AA vs Asian	<0.001
Latinx/o/a vs Asian	<0.001

© 2023 Saberi B et al. *JAMA Network Open.*

eTable 4. Characteristics of LT Recipients Who Did Not Have HCC Accoridng to Race

	**Total**	**Asian**	**Black/AA**	**Latinx/o/a**	**White**	**p-value**
**Characteristics**	**N**=43079	**N**=1195	**N**=3641	**N**=6011	**N**=32232	
**Age, Mean (SD)**	53.7 (10.9)	52.0 (11.9)	51.2 (12.1)	52.7 (11.0)	54.3 (10.7)	**<.0001**
**Female, n (%)**	16561 (38.4)	469 (39.3)	1693 (46.5)	2447 (40.7)	11952 (37.1)	**<.0001**
**ABO, n (%)**						**<.0001**
A	15696 (36.4)	305 (25.5)	858 (23.6)	1794 (29.9)	12739 (39.5)	
B	5914 (13.7)	370 (31.0)	856 (23.5)	731 (12.2)	3957 (12.3)	
AB	2258 (5.2)	130 (10.9)	198 (5.4)	179 (3.0)	1751 (5.4)	
O	19211 (44.6)	390 (32.6)	1729 (47.5)	3307 (55.0)	13785 (42.8)	
**BMI (kg/m^2), Mean (SD)**	29.2 (6.0)	25.8 (5.2)	28.6 (6.3)	29.5 (6.0)	29.3 (6.0)	**<.0001**
**Etiology, n (%)**						**<.0001**
1 (HCV)	9548 (22.2)	165 (13.8)	1262 (34.7)	1461 (24.3)	6660 (20.7)	
2 (ALD)	13302 (30.9)	237 (19.8)	579 (15.9)	1915 (31.9)	10571 (32.8)	
3 (NAFLD)	8191 (19.0)	141 (11.8)	178 (4.9)	1149 (19.1)	6723 (20.9)	
4 (HBV)	754 (1.8)	267 (22.3)	131 (3.6)	48 (0.8)	308 (1.0)	
5 (other)	11284 (26.2)	385 (32.2)	1491 (41.0)	1438 (23.9)	7970 (24.7)	
**Diabetes mellitus, n (%)**	11202 (26.0)	349 (29.2)	913 (25.1)	1844 (30.7)	8096 (25.1)	**<.0001**
**HE, n (%)**	30321 (70.4)	756 (63.3)	2243 (61.6)	4289 (71.4)	23033 (71.5)	**<.0001**
**Ascites, n (%)**	36107 (83.8)	920 (77.0)	2840 (78.0)	5160 (85.8)	27187 (84.4)	**<.0001**
**MELD Score, Mean (SD)**	26.3 (9.0)	28.0 (9.5)	27.2 (8.7)	28.3 (9.1)	25.8 (9.0)	**<.0001**
**MELD exception, Mean (SD)**	28.4 (8.1)	30.4 (8.3)	29.0 (7.9)	30.3 (8.3)	27.9 (8.0)	**<.0001**
**Creatinine (mg/dl), Mean (SD)**	1.8 (1.6)	1.9 (1.9)	2.4 (2.3)	1.9 (1.7)	1.8 (1.5)	**<.0001**
**Bilirubin (mg/dL), Mean (SD)**	10.8 (11.9)	15.2 (14.4)	12.0 (12.3)	12.8 (13.0)	10.2 (11.4)	**<.0001**
**INR, Mean (SD)**	2.1 (1.0)	2.3 (1.2)	2.2 (1.1)	2.2 (1.1)	2.1 (1.0)	**<.0001**
**Albumin (g/dL), Mean (SD)**	3.1 (0.7)	3.1 (0.8)	2.9 (0.8)	3.2 (0.8)	3.1 (0.7)	**<.0001**
**Wait Time (Days), Mean (SD)**	216.9 (467.3)	232.8 (565.5)	200.9 (445.4)	232.6 (488.6)	215.2 (461.6)	**<0.01**
Donor characteristics						
**Age (Years), Mean (SD)**	40.8 (16.0)	39.9 (16.7)	38.6 (15.3)	40.3 (16.0)	41.1 (16.0)	**<.0001**
**Female, n (%)**	17197 (39.9)	516 (43.2)	1502 (41.3)	2353 (39.1)	12826 (39.8)	**0.02**
**BMI (kg/m^2), Mean (SD)**	27.9 (6.5)	26.8 (5.9)	27.5 (6.3)	27.4 (6.2)	28.1 (6.6)	**<.0001**
**Race, n (%)**						**<.0001**
White	27977 (64.9)	667 (55.8)	2221 (61.0)	3173 (52.8)	21916 (68.0)	
African American	7777 (18.1)	198 (16.6)	855 (23.5)	913 (15.2)	5811 (18.0)	
Latinx/o/a	5522 (12.8)	232 (19.4)	425 (11.7)	1482 (24.7)	3383 (10.5)	
Asian	1009 (2.3)	66 (5.5)	2.0 (2.4)	209 (3.5)	647 (2.0)	
Other	794 (1.8)	32 (2.7)	53 (1.5)	234 (3.9)	475 (1.5)	
**Total Cold Ischemic Time (h), Mean (SD)**	6.2 (2.4)	6.4 (2.4)	6.1 (2.5)	6.4 (2.5)	6.1 (2.4)	**<.0001**

© 2023 Saberi B et al. *JAMA Network Open.*

Abbreviations: ALD: alcoholic liver disease; BMI: body mass index, HBV: hepatic B virus, HCC: hepatocellular carcinoma, HCV: hepatic C virus, HE: hepatic encephalopathy, INR: international normalization ratio, MELD: model for end-stage liver disease, NAFLD: non-alcoholic fatty liver disease, PVT: portal vein thrombosis; SD: standard deviation, TIPS: transjugular intrahepatic shunt

© 2023 Saberi B et al. *JAMA Network Open.*

eTable 5. Comparison of Post-LT Survival in Patients With HCC in Era 1 According to Race

**Comparison**	**p-values**
Across all 4 groups	<0.0001
White vs Black/AA	0.0004
White vs Latinx/o/a	0.003
White vs Asian	<0.0001
Black/AA vs Latinx/o/a	<0.0001
Black/AA vs Asian	<0.0001
Latinx/o/a vs Asian	0.08

© 2023 Saberi B et al. *JAMA Network Open.*

eTable 6. Comparison of Post-LT Survival in Patients With HCC in Era 2 According to Race

**Comparison**	**p-values**
Across all 4groups	0.001
White vs Black/AA	0.003
White vs Latinx/o/a	0.58
White vs Asian	0.02
Black/AA vs Latinx/o/a	0.03
Black/AA vs Asian	0.0002
Latinx/o/a vs Asian	0.02

© 2023 Saberi B et al. *JAMA Network Open.*

eFigure. Trend of Proportion of Liver Transplants

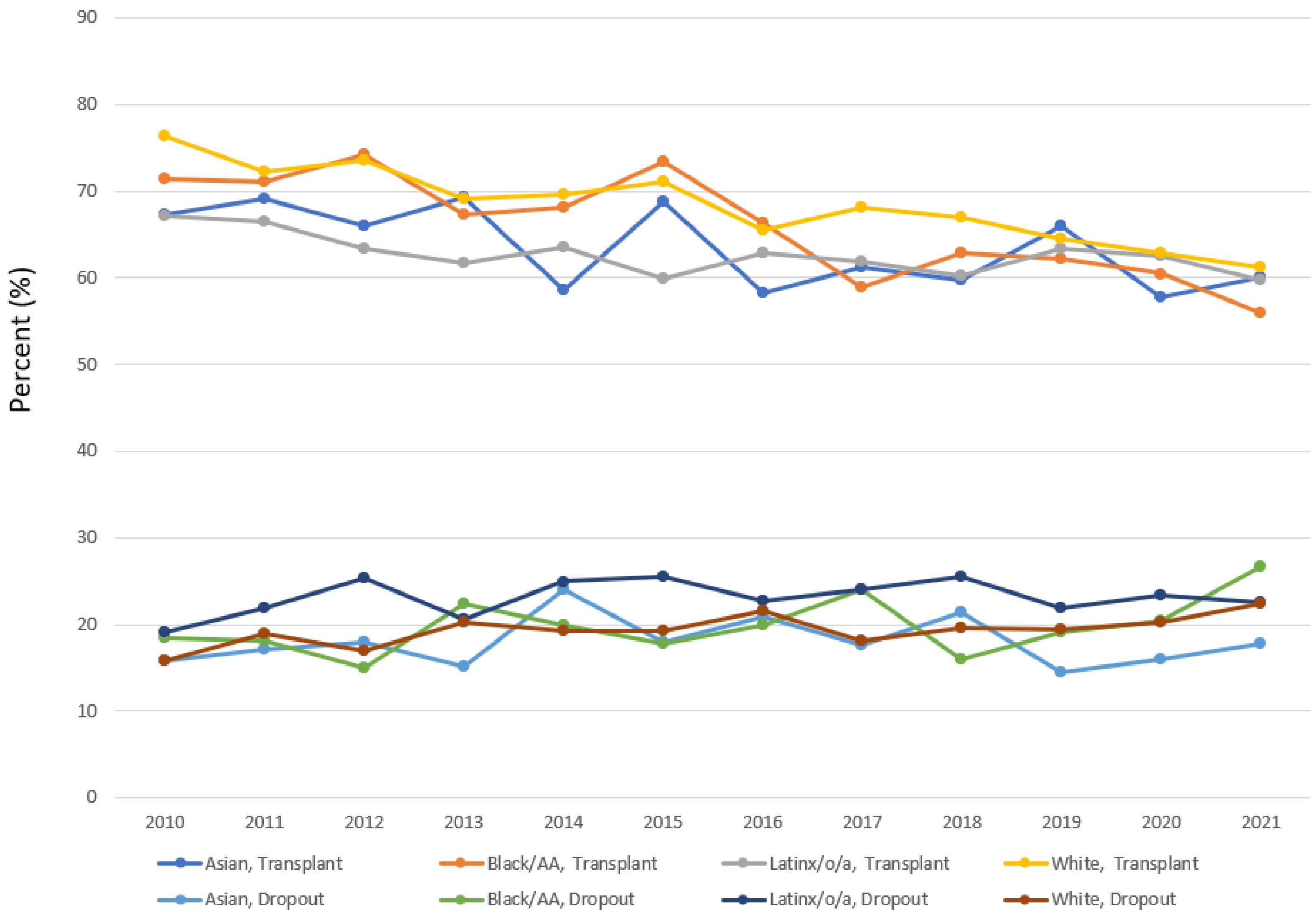

eFigure 1A. Trend of proportion of liver transplant among HCC waitlisted candidates by race and Trend of proportion of drop-out among HCC waitlisted candidates by race. Based on OPTN data as of July 1st 2022

© 2023 Saberi B et al. *JAMA Network Open.*

eFigure 1B. Trend of proportion of liver transplant among non-HCC waitlisted candidates by race and Trend of proportion of drop-out among non-HCC waitlisted candidates by race. Based on OPTN data as of July 1, 2022.

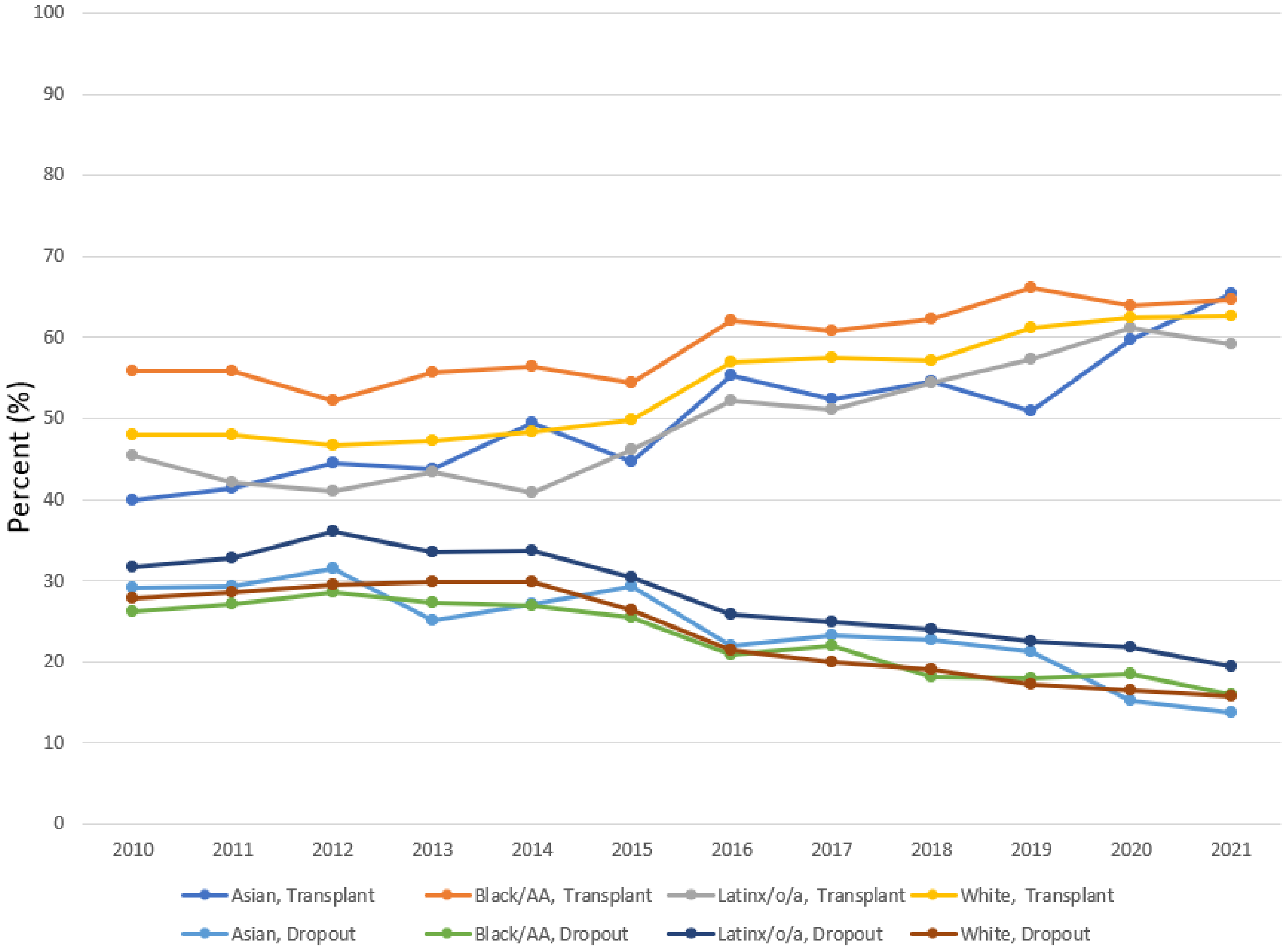

eFigure 1B. Trend of proportion of liver transplant among non-HCC waitlisted candidates by race and Trend of proportion of drop-out among non-HCC waitlisted candidates by race. Based on OPTN data as of July 1st 2022

© 2023 Saberi B et al. *JAMA Network Open.*
