# Supplementary material for: Racial Disparities in Candidates for Hepatocellular Carcinoma Liver Transplant After 6-Month Wait Policy Change
Source: JAMA Netw Open. 2023 Nov 2;6(11):e2341096. doi: 10.1001/jamanetworkopen.2023.41096 (PMC10623194; doi:10.1001/jamanetworkopen.2023.41096)
Supplement: Supplement 2. — Data Sharing Statement [file jamanetwopen-e2341096-s002.pdf]

Data Sharing Statement

Saberi. Racial Disparities in Candidates for Hepatocellular Carcinoma Liver Transplant After 6-Month Wait Policy Change. *JAMA Netw Open*. Published November 02, 2023. doi:10.1001/jamanetworkopen.2023.41096

Data
Data available: No

Additional Information
Explanation for why data not available: UNOS does not allow data sharing. However, this is a publicly available database that can be requested through UNOS/OPTN.
